# Supplementary material for: MetaRibo-Seq measures translation in microbiomes
Source: Nat Commun. 2020 Jun 29;11:3268. doi: 10.1038/s41467-020-17081-z (PMC7324362; doi:10.1038/s41467-020-17081-z)
Supplement: Supplementary file 10 — Supplementary Data 7 [file 41467_2020_17081_MOESM10_ESM.zip › File2/Confidence_High_Taxonomy/189040_out.krona.html]

Javascript must be enabled to view this page.

members
magnitude
magnitudeUnassigned
count
unassigned
taxon
rank

189040\_out

2

superkingdom
2
2

2
phylum
1239

91061
class
2

186826
order
2

2
family
1300


SRS055177\_contig\_number\_contig-100\_19939.115341SRS098616\_contig\_number\_2081
2
genus
1301
